# Pharmacists and Physicians Hand in Hand

**Published:** 1907-12

**Authors:** Solomon Solis-Cohen

**Affiliations:** Philadelphia; Chairman of the Delegation from the Section of Pharmacology and Therapeutics of the American Medical Association to the meeting of the American Pharmaceutical Association


### Pharmacists and Physicians Hand in Hand.

By SOLOMON SOLIS-COHEN, M.D., Philadelphia.

Chairman of the Delegation from the Section of Pharmacology and Therapeutics of the American Medical Association to the meeting of the American Pharmaceutical Association.

For the last few years pharmacists and physicians working hand in hand, have set themselves to change some of their mutual errors and mistakes of the past. It lies not in the mouth of the physician to reproach the pharmacist nor in the mouth of the pharmacist to reproach the physician. We have erred mutually, we have erred together, and we are determined to redeem ourselves together. The mere trade in patent medicines, in frauds and fakes, the deceits of all kinds, need not concern us. There are crimes outside of the ranks of medicine and outside of the ranks of pharmacy and we are not starting off on a general reform expedition. There are other organisations and other agencies for that purpose, but the movement to make the drugs—whether the product of the manufacturing houses or the product of the individual pharmacist—which are dispensed over the counter, upon our prescriptions, what they purport to be is one in which you and we have a common interest, and in which our patients have the greatest interest of all. I recognise and you recognise—we must recognise—that in the general progress of science and the general advance of discovery, and the general progress of the arts of manufacturing and preparation of crude pharmaceutics there is abundant room for large manufacturing houses which devote themselves to specialties of various kinds.

MANUFACTURING PHARMACY.

For example, how can the individual pharmacist undertake to prepare and supply the great group of animal extracts and serums, which now have such a large part in the therapeutics of today? And so even with various galenicals, alkaloids and the like. There are many things which the retail pharmacist cannot do as well as that establishment which possesses the proper facilities and which is thoroughly organised to do well on a large scale what can only be done imperfectly on a small scale. We all recognise that, and the American Medical Association has taken steps, individual physicians have taken steps, to place themselves in proper relation with the great manufacturing houses, which are a credit to American Pharmacy and to American business. We want to have the most cordial relations with them, so that these firms may be encouraged to prepare and offer to us for the benefit of our patients the best and purest and most definite pharmaceutical products. And yet, after all, there is a place, and there must be a place always for the individual pharmacist—the retail druggist, call him by whatever name you please; for the individual who practices as a scientific man the profession of pharmacy.

